# Errors of Fixed QT Heart Rate Corrections Used in the Assessment of Drug-Induced QTc Changes

**DOI:** 10.3389/fphys.2019.00635

**Published:** 2019-06-18

**Authors:** Katerina Hnatkova, Jose Vicente, Lars Johannesen, Christine Garnett, Norman Stockbridge, Marek Malik

**Affiliations:** ^1^National Heart and Lung Institute, Imperial College London, London, United Kingdom; ^2^Division of Cardiovascular and Renal Products, Office of New Drugs, Center for Drug Evaluation and Research, U.S. Food and Drug Administration, Silver Spring, MD, United States

**Keywords:** drug safety, drug-induced QTc changes, heart rate correction of QT interval, Fridericia QTc formula, Framingham QTc formula, subject-specific QTc corrections

## Abstract

The accuracy of studies of drug-induced QTc changes depends, among others, on the accuracy of heart rate correction of QT interval. It has been recognized that when a drug leads to substantial heart rate changes, fixed universal corrections cannot be used and that alternative methods such as subject-specific corrections established for each study participant need to be considered. Nevertheless, the maximum heart rate change that permits use of fixed correction with reasonable accuracy has not been systematically investigated. We have therefore used full QT/heart-rate profiles of 751 healthy subjects (mean age 34.2 ± 9.6, range 18–61 years, 335 females) and compared their subject-specific corrections with 6 fixed corrections, namely Bazett, Fridericia, Framingham, Hodges, Rautaharju, and Sarma formulae. The comparison was based on statistical modeling experiments which simulated clinical studies of *N* = 10 or *N* = 50 female or male subjects. The experiments compared errors of ΔQTc intervals calculated as differences between QTc intervals at an initial heart rate (in the range of 40 to 120 beats per minute, bpm) and after a heart rate change (in the range from −20 to +20 bpm). The experiments also investigated errors due to spontaneous heart rate fluctuation and due to omission of correction for QT/RR hysteresis. In each experiment, the absolute value of the single-sided 90th percentile most remote from zero was used as the error estimate. Each experiment was repeated 10,000 times with random selection of modeled study group. From these repetitions, median and upper 80th percentile was derived and graphically displayed for all different combinations of initial heart rate and heart rate change. The results showed that Fridericia formula might be reasonable (with estimated errors of ΔQTc below 8 ms) in large studies if the heart rate does not change more than ± 10 bpm and that the errors by fixed corrections and the errors due to omission of QR/RR hysteresis are additive. Additionally, the results suggest that the variability introduced into QTc data by not correcting for the underlying heart rate accurately might have a greater impact in smaller studies. The errors by Framingham formula were practically the same as with the Fridericia formula. Other investigated fixed heart rate corrections led to larger ΔQTc errors.

## Introduction

The possibility of proarrhythmic potential of novel pharmaceutical compounds is well recognized (Guidance to industry, 2005; Vicente et al., 2016) and related considerations are an integral part of regulatory process of the approval of new drugs (Zhang et al., 2015). While novel approaches to these considerations are presently discussed (Darpo et al., 2014; Vicente et al., 2018), the assessment of drug-induced QTc interval prolongation remains a principal test to identify compounds that require further evaluation of their propensity of triggering Torsade de Pointes (TdP) tachycardia. The accuracy of the evaluation of drug-induced QTc interval prolongation depends on a number of factors including the quality of electrocardiogram (ECG) recordings, precision of their measurement and validity of pharmacokinetic / pharmacodynamic modeling (Garnett et al., 2008). When a drug changes heart rate, the assessment of QTc changes also crucially depends on the validity of methods used to correct the QT interval for the underlying heart rate (Garnett et al., 2012).

Practically since the invention of electrocardiography, the relationship between the QT interval duration and the underlying heart rate has been a subject of a large number of studies that proposed a variety of mathematical formulae to describe the relationship and to correct the QT interval for heart rate (Malik, 2002). More recently, it has been recognized that the QT/heart-rate relationship differs among subjects (Batchvarov et al., 2002; Malik et al., 2002) and that consequently, all fixed formulae are inaccurate to a greater or lesser extent (Malik, 2002). This is also reflected in recommendations in a white paper suggesting that in the presence of obvious underlying heart rate changes, fixed formulae cannot be relied on and that the individuality of the QT/heart-rate relationship needs to be taken into account (Garnett et al., 2012).

Nevertheless, the extent of the drug-induced heart rate changes that still allows fixed correction formulae to be used has not been systematically investigated. Discussions of whether alternative methods to account for changes in heart rate need to be used once the heart rate changes on average by some number of beats per minute (bpm) are not based on a systematic analysis of data. Having this knowledge gap in mind, we have conducted a statistical modeling study that compared a battery of previously proposed fixed heart rate correction formulae with individual QT/heart-rate profiles in a large population of healthy subjects.

In these statistical modeling experiments, we considered not only the absolute errors of fixed heart rate corrections but also errors of ΔQTc values representing the QTc changes for different heart rates since when calculation QTc changes, absolute correction errors in QTc estimates on baseline and on active treatment might partially cancel each other. Fixed corrections also only consider how much the QT interval changes at different heart rates but do not deal with the so-called QT/RR hysteresis, that is the assessment of how quickly QT interval changes after heart rate changes (Malik et al., 2008b; Gravel et al., 2018). We have therefore also included modeling experiments that combined the ΔQTc errors due to heart rate change with errors caused by the omission of QT/RR hysteresis correction.

## Materials and Methods

As explained further in more detail, of all the different previously published correction formulae, we selected 6 that were most representative of different types of QT/heart-rate curvatures. We compared their correction performance with that of the curvilinear models of intra-subject QT/heart-rate relationship (Malik et al., 2013). We obtained these subject-specific data from a previously published population healthy subjects in whom repeated accurate ECG measurements were available (Malik et al., 2016). In the statistical modeling experiments, we also used previously published data to estimate spontaneous heart rate instability and QT/RR hysteresis effects.

### Fixed QT Heart Rate Corrections

Although the QT interval duration depends on the underlying heart rate rather than on the duration of the preceding cardiac cycle (Franz et al., 1988), it became customary to correct the QT interval duration for the duration of the RR interval representing the underlying heart rate. Therefore, our study also used the conversion of heart rate changes into RR interval changes allowing to employ previously published corrections in their original form.

The following six correction formulae were selected:

Bazett (Bazett, 1920)
  QTc = QT/$\sqrt{\text{RR}}$
Fridericia (Fridericia, 1920)
  QTc = QT/$\sqrt[{3}]{\text{RR}}$
Framingham (Sagie et al., 1992)
  QTc = QT + 0.154(1 − RR)
Hodges (Hodges et al., 1983)
  QTc = QT + 0.00175(HR − 60)
Rautaharju (Rautaharju et al., 1990)
  QTc = QT + 0.24251 − 0.434^∗^*e*^−0.0097^∗^HR^
Sarma (Sarma et al., 1984)
  QTc = QT - 0.04462 + 0.664^∗^*e*^−2.7^∗^RR^

where QTc, QT, and RR are ECG intervals measured in seconds and HR is the heart rate in bpm.

**FIGURE 1:**
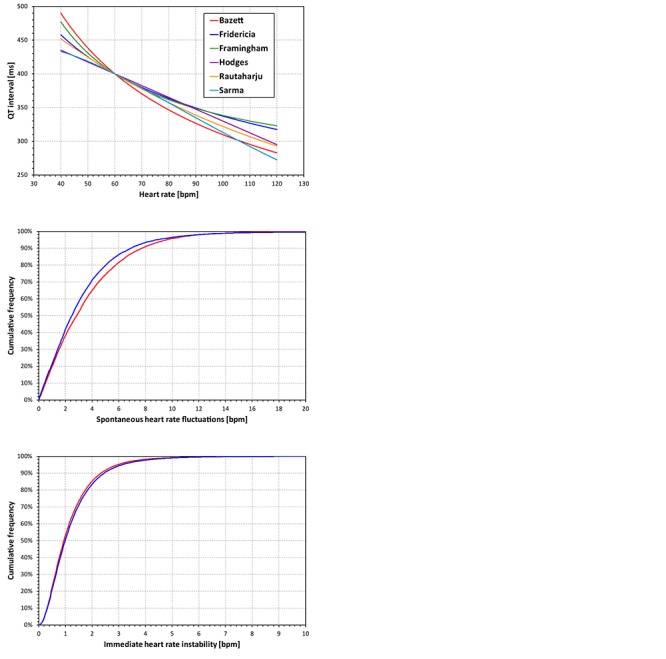
The top panel shows curvatures QT/heart-rate assumed by the six fixed heart rate correction formulae for a subject in whom QT = 400 at heart rate of 60 bpm. The middle and the bottom panel show the cumulative distribution of spontaneous heart rate fluctuations and of immediate heart rate instability, respectively (see the text for details). The red and blue lines correspond to female and male subjects, respectively. Note that the observed distributions of both spontaneous heart rate fluctuations and of immediate heart rate instability were symmetrical in terms of showing the corresponding distributions of both positive and negative values. Therefore, for simplicity, only one half of the distributions is presented showing the positive values.

The Bazett and Fridericia formulae were selected because of their frequent use, the Framingham and Hodges formulae relate QT interval linearly to the RR interval and to the heart rate, and similarly, the Sarma and Rautaharju formulae relate QT interval exponentially to the RR interval and to the heart rate. Examples of the curvatures of these formulae are shown in the top panel of Figure 1.

For the purposes of describing the experiments with these fixed formulae, we shall use the symbol *QTc*_■_(QT,HR) which will mean the results of a fixed formula applied to QT interval corrected for heart rate HR. Where necessary, we use subscripts B, F, Fm, H, R, and S to denote formulae from Bazett to Sarma, respectively.

### Population Data

The study used data that have previously been used in conceptually different investigations (Malik et al., 2016). In brief, the data originated from 7 thorough QT studies. Pooled together, these studies investigated 751 individuals, mean ± standard deviation (SD) age 34.18 ± 9.56 years, range 18–61, 335 females. All were healthy subjects with normal physical investigation and normal screening ECG. The source studies were all approved by relevant ethics authorities and all subjects gave written informed consent in accordance with the Helsinki declaration and giving permission for their data to be used in scientific research. The original studies investigated different novel pharmaceuticals, but since we used only anonymised drug-free data, the character of the investigated drugs is irrelevant. For the same reason, no separate ethics clearance of the present investigation was required as per the local legislation.

In each subject, the source studies obtained repeated day-time 12-lead Holter recordings. During drug-free days, multiple QT interval and hysteresis-corrected RR intervals were measured scanning broad ranges of heart rates in each subject. A total of 897,570 ECG measurements (QT interval + hysteresis corrected RR interval, range 321–1560 measurements per subject) were made. Using previously described procedures (Malik et al., 2008a, 2012) the QT interval measurements were made in representative median complexes derived from 10-s ECG segments. The accuracy of each measurement was ensured by repeated visual verifications and, where necessary, manual corrections.

### Individual QT/RR Patterns

In the pooled study population, the heart rates (corresponding to hysteresis corrected RR intervals (Malik et al., 2008b)) at which QT interval were measured ranged between 32.0 and 164.3 bpm, (33.9–164.3 bpm in females, 32.0–151.3 bpm in males). The within-subject ranges (max–min) of heart rates at which QT intervals were measured were (mean ± SD) 61.1 ± 12.7 bpm and 57.8 ± 12.9 bpm wide in females and males, respectively.

Using previously published technique (Malik et al., 2013), an individual-specific QT/RR curvilinear regression in the form

$$\text{QT}=\alpha +\frac{\beta }{\gamma }({\text{RR}}^{\gamma }-1)+\varepsilon$$

was fitted to the QT and RR interval data in each subject. In this form, QT and RR intervals are in seconds (RR representing the hysteresis-corrected underlying heart rate at which the QT interval was measured), ε are normally distributed zero-centered errors, and parameters α, β, and γ represent the individual-specific values of the central rate-independent QT value, and of the slope and curvature of the QT/RR relationship, respectively. This regression formula leads to individual-specific QT correction in the form:

$$\text{QTc}=\text{QT}+\frac{\beta }{\gamma }(1-{\text{RR}}^{\gamma })$$

These curvilinear regressions fitted the individual data tightly. The mean regression residuals (i.e., the standard deviations of the individually corrected QTc values) were (mean ± SD) 5.68 ± 1.10 ms and 5.33 ± 1.10 ms in females and males, respectively.

The RR intervals representing the hysteresis-corrected underlying heart rate at which the QT interval was measured were obtained using the previously published exponential decay model (Malik et al., 2008b): If QT interval reading is preceded by RR interval sequence ${\left\{{\mathrm{RR}}_{\text{i}}\right\}}_{\text{i}=0}^{\text{N}}$ (*RR*_0_ closest to the QT measurement), and $\Lambda (k)=\sum_{\text{i}=0}^{\text{k}}{\mathrm{RR}}_{\text{i}}$, the exponential hysteresis model suggests correcting the QT interval for ${\mathrm{RR}}^{\prime }=\sum_{\text{i}=0}^{\text{N}}\omega_{\text{i}}{\mathrm{RR}}_{\text{i}}$, where for each *j* = 0, ⋯, *N*, $\sum_{\text{i}=0}^{\text{j}}\omega_{\text{i}}=\left(1-e^{-\lambda \Lambda (j)/\Lambda (N)}\right)/\left(1-e^{-\lambda }\right)$ where the coefficient λ characterizes the subject-specific time constant, i.e., the speed with which QT interval adapts to a change in the underlying heart rate. In the data available for the experiments described here, 5-min histories of all QT interval measurements were available, that is Λ (*N*) ≅ 300s.

The curvilinear QT/RR regression modeling allowed us to estimate reliably the QT interval value for different heart rates. That is, for any given heart rate, the representative RR interval could be calculated and, together with parameters α, β, and γ, turned into the QT interval in the given subject at the given heart rate. For the purposes of describing the computations made in this study, we shall use the symbol *QT*(HR) meaning the QT interval in a given subject at heart rate HR (i.e.,

$$\mathrm{QT}(\text{HR})=\alpha +\frac{\beta }{\gamma }[{\left(60/\text{HR}\right)}^{\gamma }-1],$$

where the coefficients α, β, and γ correspond to the given subject and the heart rate value HR is in bpm). This means that for every subject and every heart rate HR, the individual-specific correction formula applied to *QT*(HR) always leads to the same constant value (= α). Indeed, this is the basic principle of individual-specific heart rate correction (Garnett et al., 2012; Malik, 2018). In the description of the computations, we shall denote this constant value by *QTcI*.

### Basic Evaluation of Fixed Heart Rate Corrections

A simple evaluation of the accuracy of fixed corrections can be based on the comparison of their results with the results of the individual-specific corrections. That is, for different values of heart rate h, we evaluated the differences between *QTcI* and *QTc*_■_(QT(h),h) in each study subject. Subsequently, for each fixed correction formula, these differences were statistically summarized over the complete study populations. In these summaries, we distinguished female and male study subjects because of the known sex differences in the QT/heart-rate patterns (Linde et al., 2018).

The differences *QTc*_■_(QT(h), h) − QTcI were evaluated for h between 40 and 120 bpm in 0.1 bpm steps.

### Statistical Modeling Experiments

The errors of fixed corrections at different heart rates evaluated over the complete population are potentially misleading in several ways. For the purposes of a more appropriate assessment, we performed three sets of statistical modeling experiments.

#### Experiments Evaluating QTc Errors Due to Heart Rate Instability

The first problem of the simple evaluation of the differences between fixed corrections and *QTcI* is the assumption that the very same heart rate might be considered in all subjects. This does not correspond to the reality of clinical studies in which, even during baseline conditions, subjects show short-term variable heart rates. Therefore, using previously published data of 73 thorough QT studies in which 747,912 ECGs were measured in 6,786 healthy subjects (Malik et al., 2016), we obtained a distribution of inter-subject heart rate differences at the same study time-points. That is, in all these studies and subjects, we considered drug-free time-points of each study when the ECGs were measured under the same conditions (e.g., supine, resting, and fasting). In each subject and at each time-point, heart rate measurements were made. Subsequently, we obtained the distribution of heart rate changes between different (drug-uninfluenced) time-points within the same subject. This distribution is shown in the middle panel of Figure 1. We shall call this the distribution of the spontaneous heart rate fluctuations. It shows how much the heart rate fluctuates in the same subject between time-points of the same character (e.g., when the subject is placed repeatedly into supine resting position during fasting morning hours of the same day).

Subsequently, in the first type of statistical models, we considered a sub-population of *N* subjects and for each of these subjects and for different values of heart rate h, we evaluated the differences between *QTcI* and

$${\mathrm{QTc}}_{■}(\text{QT}(\text{h}+\varepsilon ),\text{ h}+\varepsilon )$$

where ε was a random number derived from the distribution the spontaneous heart rate fluctuations (different random numbers from the distribution were used in different individual comparisons).

If the QTc values were accurate (i.e., if the individual-specific heart rate corrections were used instead of fixed corrections) each the differences would be equal to zero. Therefore, to characterize the results of the comparison in *N* subjects, we calculated the largest absolute difference |*QTcI* − *QTc*_■_(QT(h+*ε*),h+*ε*)| after excluding the lowest and highest 10% of the *QTcI* − *QTc*_■_(QT(h+*ε*),h+*ε*) differences.

#### Experiments Evaluating QTc Errors Due to Drug-Induced Heart Rate Changes

The accuracy of fixed corrections also needs to consider situations of heart rate changes. As explained previously, in each individual, the subject-specific heart rate correction results in the same value of *QTcI* when correcting QT(h) for the heart rate h irrespective of the value of h. Hence, if the heart rate changes, there would be no difference in subject-specific heart rate correction between QT(h) corrected for h and QT(h+δ) corrected for h+δ. It is therefore appropriate to investigate the difference in fixed QTc values for the same scenario.

In addition to this, the considerations of spontaneous heart rate fluctuations also need to be incorporated. We therefore considered a sub-population of *N* subjects and for each of these subjects, evaluated the difference

$$\begin{array}{l} {\mathrm{QTc}}_{■}(\mathrm{QT}(\text{h}+\varepsilon_{1}),\text{ h}+\varepsilon_{1})\\ \;-{\mathrm{QTc}}_{■}(\mathrm{QT}(\text{h}+\delta +\varepsilon_{2}),\text{ h}+\delta +\varepsilon_{2}) \end{array}$$

where h is an initial heart rate, δ is a (drug-induced) heart rate change, and ε_1_ and ε_2_ are random numbers derived from the distribution the spontaneous heart rate fluctuations. (Note that ε_1_ and ε_2_ may be positive or negative, independently of each other).

Similar to the previous type of statistical experiments, we have characterized the result of this calculation in *N* subjects by evaluating the largest absolute difference among the *QTc*_■_ values after excluding the lowest and highest 10% of the differences.

#### Experiments Evaluating QTc Errors Due to QT/RR Hysteresis Omission

Finally, the effects of QT/RR hysteresis also need to be considered. The previous type of experiments corresponds to the situation where each QT interval is corrected for a stable underlying heart rate. In practice, HR shows short-term variability, and the adaptation of QT to HR is not instantaneous, resulting in QT/RR hysteresis (Malik et al., 2016).

This error is worst when the QT interval duration is corrected for the preceding RR interval, and underlying heart rate can only be fully assessed over periods much longer than typical 10-s ECG recordings. Similar to the distribution of the spontaneous heart rate fluctuations, we have estimated the differences between the heart rate underlying the QT interval and the simultaneously measured heart rate by studying the heart rate differences between closely coupled replicate QT/RR measurements of the same time-points in the previously published data of 73 thorough QT studies (Malik et al., 2016). That is, in all these studies and subjects, we considered study drug-free time-points corresponding the same condition (e.g., supine, resting, and fasting). In each subject and at each time-point, multiple heart rate (or RR interval) measurements were made and we obtained the distribution of heart rate discrepancies between the repeated heart rate measurements within the same subject and within the same (drug-uninfluenced) time-point. The distribution of these data is shown in the bottom panel of Figure 1 and we shall call it the distribution of immediate heart rate instability. It shows how much heart rate fluctuates between measurements during the same time-point (e.g., within the same minute while the subject is in supine resting position for the duration of the same study time-point).

Using these data, we have investigated the combined effect of fixed correction formulae together with the omission of QT/RR hysteresis correction in the following experiments: Considering a sub-population of *N* subjects, we evaluated, for each of these subjects, the difference

$$\begin{array}{l} {\mathrm{QTc}}_{■}(\mathrm{QT}(\text{h}+\varepsilon_{1}),\text{ h}+\varepsilon_{1}+\vartheta_{1})\\ \;-{\mathrm{QTc}}_{■}(\mathrm{QT}(\text{h}+\delta +\varepsilon_{2}),\text{ h}+\delta +\varepsilon_{2}+\vartheta_{2}) \end{array}$$

where h is an initial heart rate, δ is heart rate change, ε_1_and ε_2_ are random numbers derived from the distribution of the spontaneous heart rate fluctuations, and ϑ_1_ and ϑ_2_ are random numbers derived from the distribution of immediate heart rate instability. Hence, the coefficients ϑ_1_ and ϑ_2_ represent the differences between the heart rates that underlie the QT intervals and the heart rates for which the QT interval is corrected. (Note that again, ε_1_ and ε_2_ and similarly ϑ_1_ and ϑ_2_ may be positive or negative, independently of the other parameters).

To characterize the result of this calculation in *N* subjects, we have again used the largest absolute difference among the *QTc*_■_ values after excluding the lowest and highest 10% of the *QTc*_■_ differences.

### Organization of Experiments

All the experiments were performed by varying the value of h from 40 bpm to 120 bpm in 0.1 bpm steps. The experiments that included the modeled heart rate changes were performed by additionally varying the value of δ from −20 to +20 bpm, again in 0.1 bpm steps (that is, in the experiments of modeled heart rate changes, we used 801 × 401 = 321,201 combinations of h and δ-values).

All experiments were also repeated for *N* = 50 and *N* = 10, to approximate the situations of standard thorough QT studies (Zhang and Machado, 2008) and of small early clinical investigations that have also been proposed for the assessment of drug-induced QTc changes (Darpo et al., 2014).

For both *N* = 50 and *N* = 10, we repeated the selection of the sub-populations 10,000 times, each time selecting the sub-population randomly from the pool of study subjects, repeating the same process for both female and male study populations. In each repetition of each experiment and in each setting of different h and δ-values, the coefficients ε_1_and ε_2_, and, in the last type of experiments, the coefficients ϑ_1_ and ϑ_2_ were repeatedly randomly generated from the relevant distributions. Mersenne twister pseudorandom number generator was used for all random selections (Matsumoto and Nishimura, 1998).

### Statistical Summaries and Results Presentation

As stated, the basic evaluations of the fixed corrections, i.e., those based on differences *QTc*_■_(QT(h),h)-*QTcI*, were performed for female and male subjects separately. For each value of h, and for each sex-group, we calculated the median, the inter-quartile range, and ranges between 10th and 90th, and between 5th and 95th percentiles of the differences in the complete sex-group. The results were presented graphically.

For each set of experiments of QTc errors due to heart rate instability, i.e., for experiments with *N* = 50 and *N* = 10 of female and male subjects, the repetition of random subject selections provided 10,000 experiment characteristics for each heart rate value of h. Of these, we again calculated the median, the inter-quartile range, and ranges between 10th and 90th, and between 5th and 95th percentiles. There dependencies on h were again presented graphically.

Similarly, the repetitions of experiments of QTc errors due to drug-induced heart rate changes and due to QT/RR hysteresis omission provided 10,000 experiments characteristics for each experiment setting and for each combination of initial heart rates h and heart rate changes δ. Of these, we calculated the median and, for the purposes of power sample estimates, also the 80th percentile. The values of these medians and 80th percentiles were used to generate color contour maps. The values of these medians and 80th percentiles were also averaged for different regions of h and δ combinations.

## Results

The characteristics of the subject-specific QT/RR curvilinear models that were used in the computational experiments are summarized in Table 1.

**Table 1:** The table shows the summaries (mean ± standard deviation) of QT/RR characteristics of the population used in the computational experiments.

	Female	Male
N	335	416
QTcI interval (ms)	420 ± 14	401 ± 12
QT/RR hysteresis constant (95% adaptation) (s)	112 ± 20	117 ± 21
QT/RR slope (parameter β)	0.161 ± 0.033	0.141 ± 0.026
QT/RR curvature (parameter γ)	0.571 ± 0.703	0.730 ± 0.728

*QT/RR hysteresis time constants λ were recalculated into the time intervals needed for the QT interval to reach 95% adaptation after a heart rate change. Note that while the QTcI values and slope and curvature parameters β and γ were used in the computational experiments, the hysteresis time constants λ were only used during the intra-subject optimization of the slope and curvature parameters.*

Altogether, with all parameter combinations, the study involved more than 7.71 × 10^10^ experiments with random selections of 10 or 50 subjects.

### Population Performance of the Correction Formulae

**FIGURE 2:**
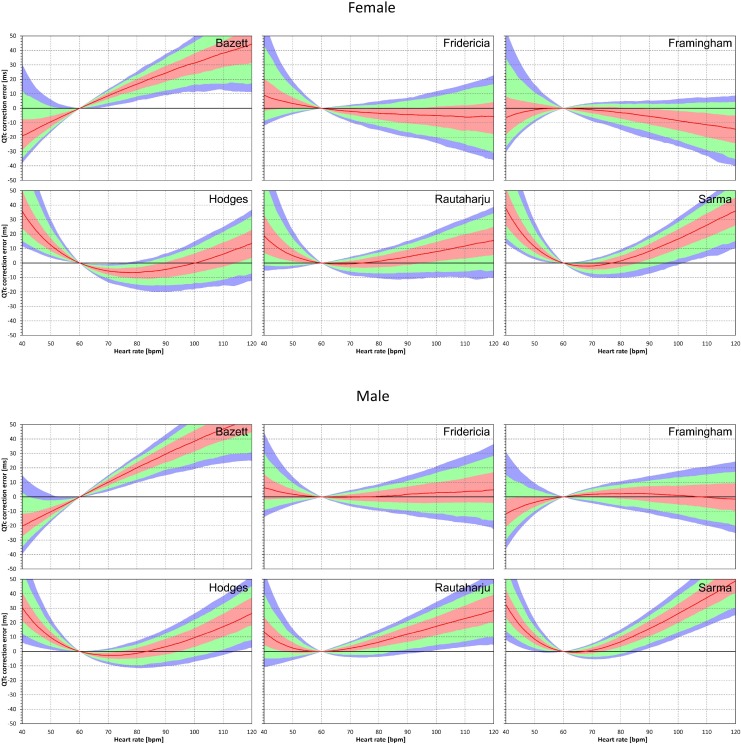
Population QTc errors of fixed heart rate correction formulae. For each formula and for each heart rate 40 to 120 bpm, the figure shows the distribution of the differences between QTc by the formula and subject-specific QTcI values. The red lines are the population medians. The pink, light green, and light blue bands show the inter-quartile ranges, and the ranges 10th to 90th, and 5th to 95th population percentiles, respectively. The top and bottom parts of the figure show the results in female and male subjects, respectively.

Population errors of the different fixed correction formulae are shown in Figure 2. By definition, all the formulae as well as the subject-specific corrections do not make any distinction between the uncorrected QT interval and corrected QTc interval at heart rate of 60 bpm. No errors of fixed formulae are therefore found at this heart rate. Nevertheless, the more the heart rate differs from 60 bpm the wider spread of errors is seen.

The median errors of QTc_F_ and QTc_Fm_ are reasonably close to zero (especially in the sub-population of male subjects) but even for these formulae, substantial over- and under-correction is seen at both slow and fast heart rates. Also, the median value of the error might correspond to different subjects at different heart rates. Similarly, the lower and higher percentiles are bound to be derived from different subjects at different heart rates.

**FIGURE 3:**
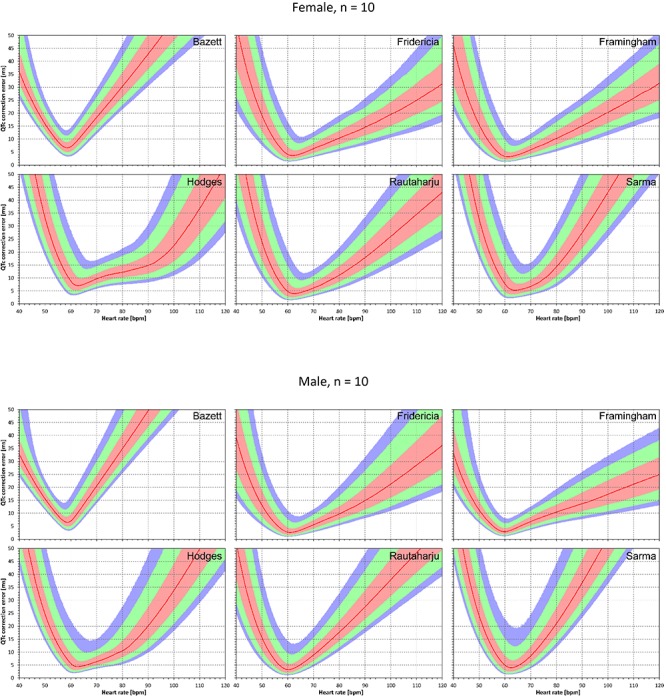
For each of the fixed correction formulae and for each heart rate 40 to 120 bpm, the figure shows the absolute QTc errors due to heart rate instability (see the text for details). The top and bottom part of the figure correspond to experiments with *N* = 10 female and male subjects, respectively. The red lines are the medians of the absolute QTc errors in 10,000 repeated experiments. The pink, light green, and light blue bands show the inter-quartile ranges, and the ranges 10th to 90th, and 5th to 95th percentiles of the repeated experiments, respectively.

Similar results are seen in Figure 3, 4 that show the evaluation of experiments evaluating QTc errors due to heart rate instability. Since heart rate instability makes the heart rates in a group of subjects always at least partially different from 60 bpm, the absolute error characteristics of these experiments are always positive. Again, the figures show that the more remote the heart rate is from 60 bpm, the more substantial absolute errors are noted. Also, not surprisingly, while the profile of the error characteristics is similar for *N* = 50 and *N* = 10, the width of the percentile bands is wider if fewer subjects are investigated (that is, the fewer subjects in the experiment, the greater possibility of more substantial errors of the fixed corrections applied to the experiment population).

**FIGURE 4:**
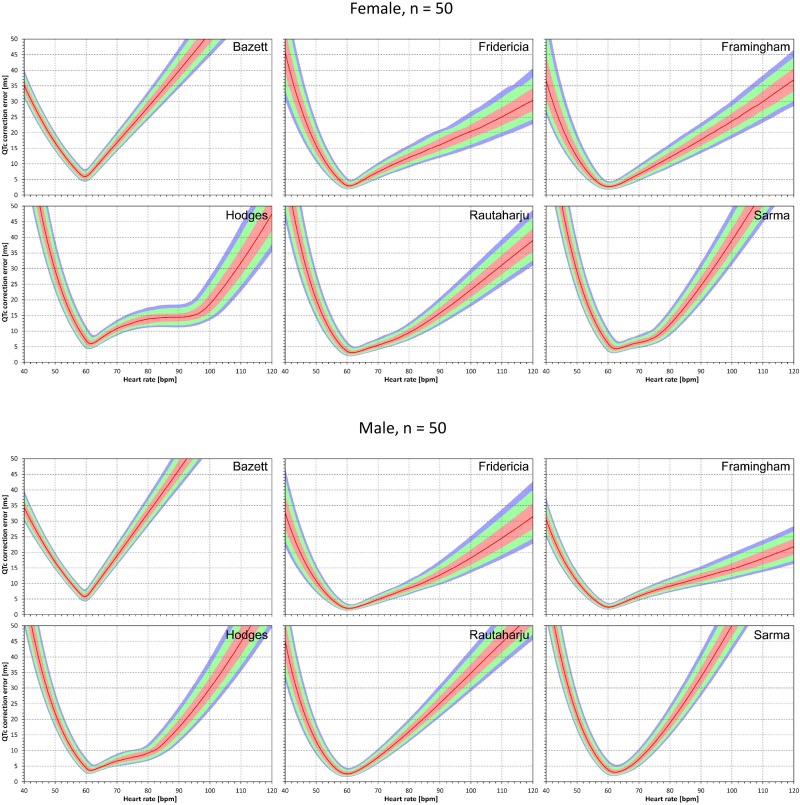
The results of absolute QTc errors due to heart rate instability shown for *N* = 50 female and male subjects. The layout of the figure is the same as in Figure 3.

### Correction Accuracy Without QT/RR Hysteresis Influence

**FIGURE 5:**
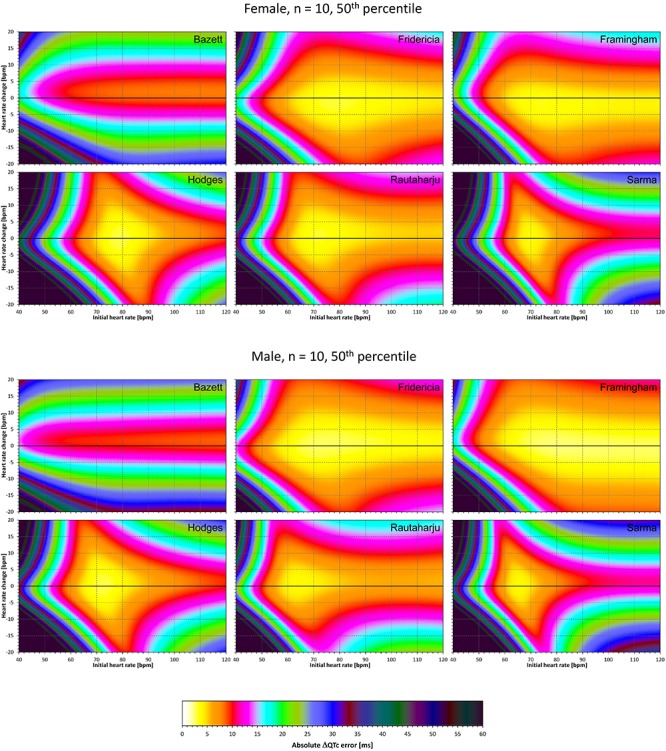
For each of the fixed correction formulae and for each combination of initial heart rate (between 40 and 120 bpm) and each heart rate change (between –20 to +20 bpm), the figure shows the absolute ΔQTc errors due to drug-induced heart rate changes (i.e., the characteristics of the corresponding statistical modeling experiments – see the text for details). The figure shows the median values of the characteristics of 10,000 experiment repetitions with *N* = 10 female subjects (top part of the figure) and with *N* = 10 male subjects (bottom part of the figure). The values are color coded according to the scale shown at the bottom of the figure.

**FIGURE 6:**
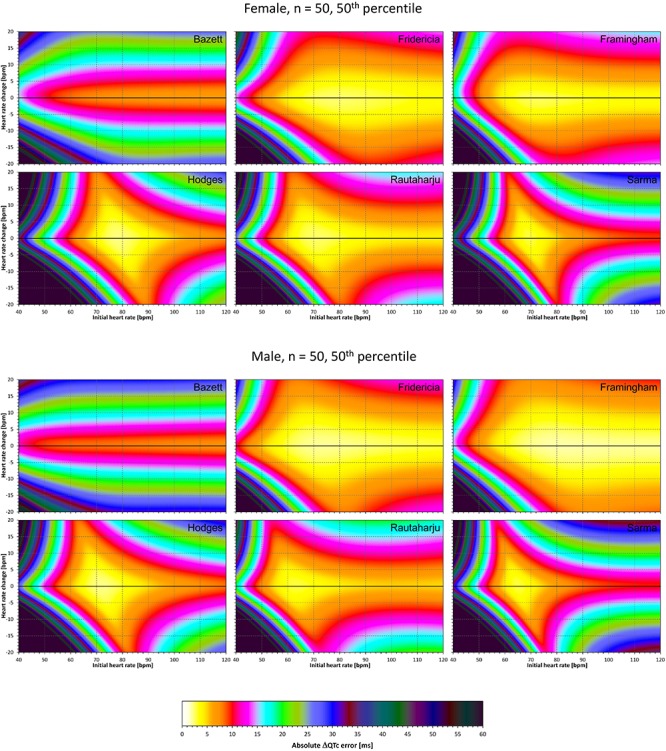
The same results as in Figure 5 but shown here for experiments with *N* = 50 female subjects (top part of the figure) and with *N* = 50 male subjects (bottom part of the figure). See Figure 5 for further details.

**FIGURE 7:**
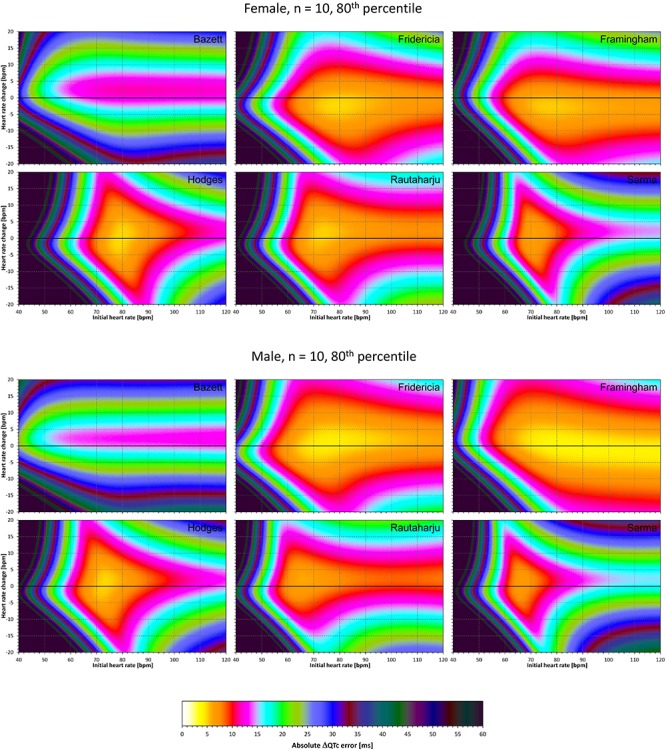
The same results as in Figure 5 but showing 80th percentile of the characteristics of 10,000 experiment repetitions with *N* = 10 female subjects (top part of the figure) and with *N* = 10 male subjects (bottom part of the figure). See Figure 5 for further details.

**FIGURE 8:**
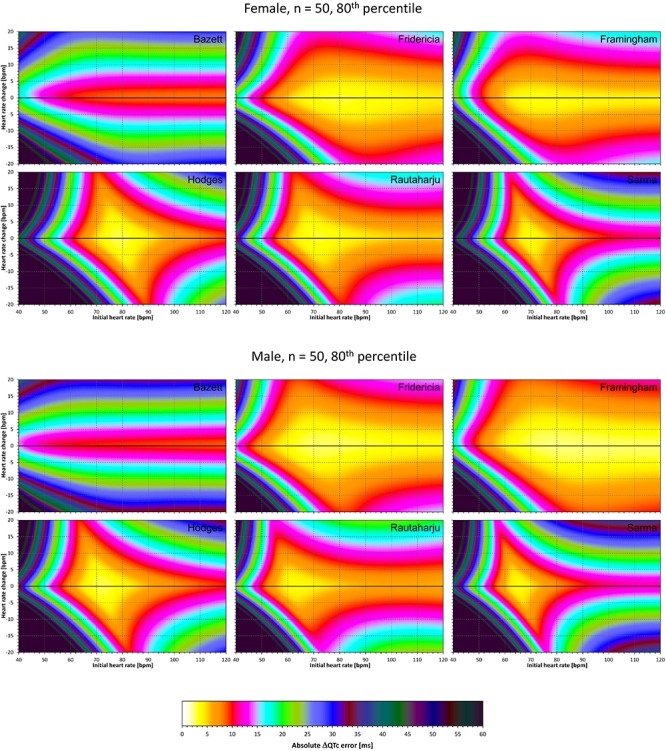
The same results as in Figure 6 but showing 80th percentile of the characteristics of 10,000 experiment repetitions with *N* = 50 female subjects (top part of the figure) and with *N* = 50 male subjects (bottom part of the figure). See Figure 5 for further details.

The results of modeling experiments evaluating QTc errors due to drug-induced heart rate changes but assuming that the effects of QT/RR hysteresis have been fully covered in the heart rate data are shown in Figure 5–8. Figure 5, 6 show the medians of error characteristics, Figure 7, 8 show their upper 80th percentiles.

Superficially, it might seem surprising that for all fixed formulae, the areas of minimum error characteristics are somewhat remote from the initial heart rate of 60 bpm. This is because the experiments investigated errors of QTc changes rather than errors of QTc values. In other words, if both

$${\mathrm{QTc}}_{■}(\mathrm{QT}(\text{h}),\text{ h}){\text{ and }\mathrm{QTc}}_{■}(\mathrm{QT}(\text{h}+\delta ),\text{ h}+\delta$$

are polluted by similar error from the true *QTcI* value, this error will cancel out when investigating the QTc effects of the heart rate change from h to h+δ. For this reason, the ΔQTc errors found in these experiments are mainly influenced by the QT/heart-rate curvatures expected by the fixed formulae. Note in Figure 2 that while the median lines are 0 at 60 bpm, they are most “flat” at somewhat different heart rates. Thus, the errors of QTc changes depend not only of the underlying heart rate change but also of the position of h and h+δ on the curvature of the correction formula.

For the same reason, the images in Figure 5–8 show rhombus-like shapes of which the long axes correspond to the values h+δ for which the curvature of the fixed formula is least departing from the true QT/heart-rate curvatures of the investigated subjects. Also, in experiments with δ = 0, it is only the combination of spontaneous heart rate fluctuations ε_1_ and ε_2_ that drive the modeled errors.

### Correction Accuracy Including QT/RR Hysteresis Influence

**FIGURE 9:**
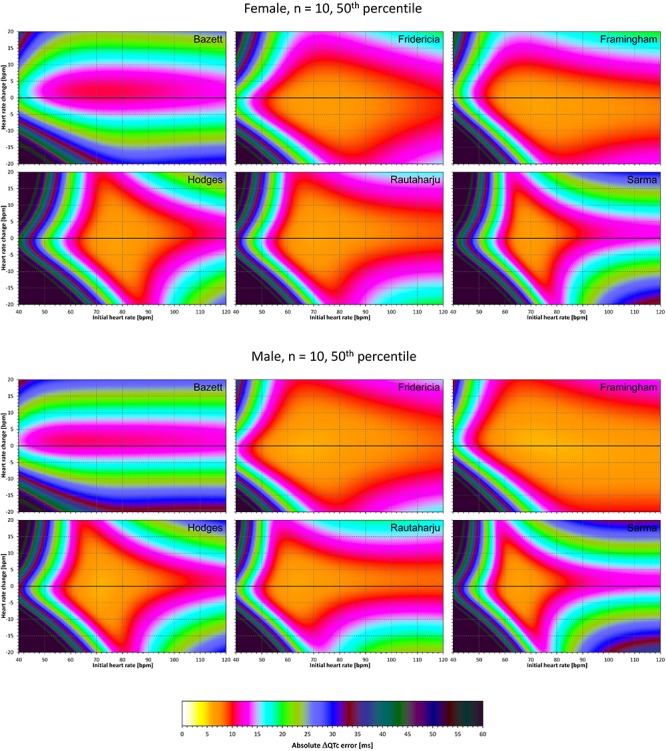
For each of the fixed correction formulae and for each combination of initial heart rate (between 40 and 120 bpm) and each heart rate change (between –20 to +20 bpm), the figure shows the absolute ΔQTc errors due to the combination of drug-induced heart rate changes and omission of QT/RR hysteresis correction (i.e., the characteristics of the corresponding statistical modeling experiments – see the text for details). The figure shows the median values of the characteristics of 10,000 experiment repetitions with *N* = 10 female subjects (top part of the figure) and with *N* = 10 male subjects (bottom part of the figure). The values are color coded according to the scale shown at the bottom of the figure.

**FIGURE 10:**
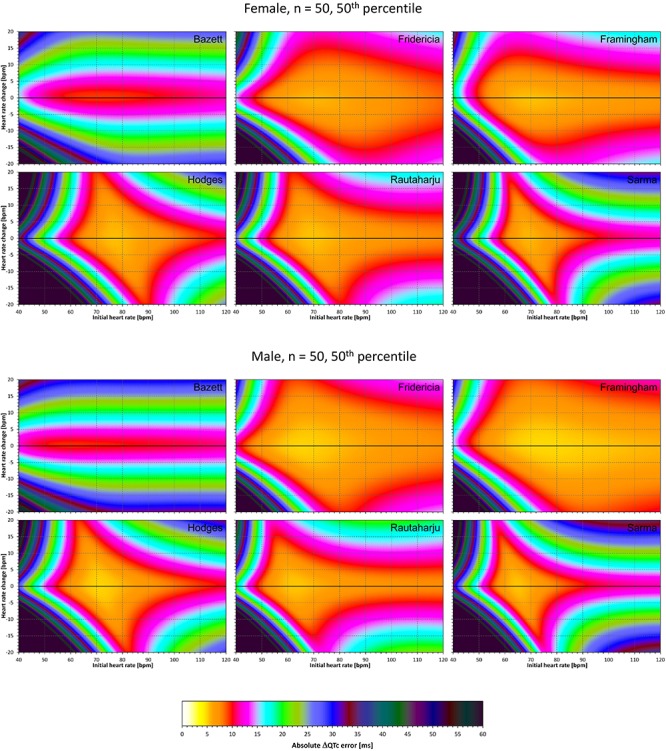
The same results as in Figure 9 but shown here for experiments with *N* = 50 female subjects (top part of the figure) and with *N* = 50 male subjects (bottom part of the figure). See Figure 9 for further details.

**FIGURE 11:**
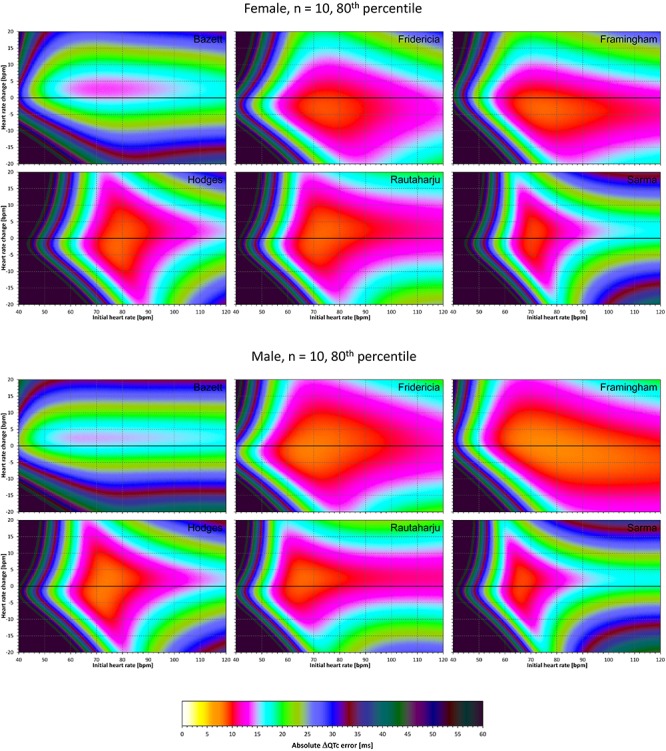
The same results as in Figure 9 but showing 80th percentile of the characteristics of 10,000 experiment repetitions with *N* = 10 female subjects (top part of the figure) and with *N* = 10 male subjects (bottom part of the figure). See Figure 9 for further details.

**FIGURE 12:**
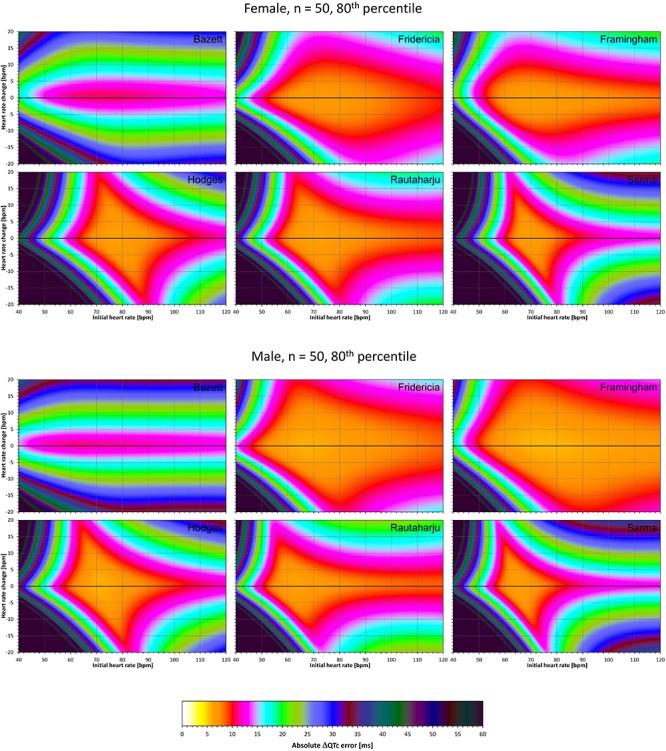
The same results as in Figure 10 but showing 80th percentile of the characteristics of 10,000 experiment repetitions with *N* = 50 female subjects (top part of the figure) and with *N* = 50 male subjects (bottom part of the figure). See Figure 9 for further details.

The results of experiments that investigated the experiments combining the effect of heart rate changes with the effect of the omission of QT/RR hysteresis are shown in Figure 9–12. Figure 9, 10 show the medians of error characteristics, Figure 11, 12 show their upper 80th percentiles.

Comparison of these figures with Figure 5–8 shows that practically irrespective of the combination of heart rate values h and h+δ, the omission of the QT/RR hysteresis adds further inaccuracy. This corresponds to previously published observations (Malik et al., 2018). When investigating the effects of omitting QT/RR hysteresis (using the same distribution data as used here) on subject-specific corrections, we observed uniform errors in the region of 3 to 7 ms for different h and δ combinations (results not shown).

### Summary of Results

To summarize the findings in Figure 5–12, Table 2 shows the averaged error characteristics (taking female and male subjects together) over the heart rate range h = 50 to 80 bpm, and for bands of heart rate changes δ bellow ± 5 bpm, between ± 5 bpm and ± 10 bpm, and between ± 10 bpm and ± 15 bpm. While the range of h = 50 to 80 bpm seems to cover reasonably the usual situations of clinical studies in healthy volunteers, very similar results (not shown) were obtained when using different ranges of h.

**Table 2:** The table shows the average values of modeled absolute ΔQTc errors (that is the experiment characteristics obtained with the sex heart rate correction formulae as shown in more detail in Figure 5–12).

		0 – ±5 bpm	±5 – ±10 bpm	±10 – ±15 bpm	0 – ±5 bpm	±5 – ±10 bpm	±10 – ±15 bpm	0 – ±5 bpm	±5 – ±10 bpm	±10 – ±15 bpm
				
N	Percentile	Bazett			Fridericia			Framingham		
**Effect of QT/RR hysteresis not included**										
10	50	11.24	16.21	22.55	4.89	7.41	11.53	5.26	8.32	13.13
50		10.35	16.06	22.37	4.07	7.12	11.18	4.28	7.15	11.48
10	80	15.81	21.14	28.15	8.51	11.87	17.11	8.86	12.99	19.37
50		12.07	18.06	24.66	5.24	8.93	14.18	5.38	8.73	13.70
**Effect of QT/RR hysteresis included**										
10	50	12.46	17.14	23.3	7.29	9.22	12.52	7.44	9.94	14.33
50		11.48	16.95	23.12	6.33	8.80	12.77	6.35	8.66	12.60
10	80	17.08	22.13	28.96	11.09	13.87	18.64	11.17	14.74	20.72
50		13.23	18.99	25.44	7.59	10.69	15.50	7.51	10.29	14.87

**N**	**Percentile**	**Hodges**			**Rautaharju**			**Sarma**		

**Effect of QT/RR hysteresis not included**										
10	50	8.67	12.88	19.53	6.02	9.03	13.94	8.81	13.12	20.17
50		7.75	13.28	21.06	5.18	9.13	15.10	7.86	13.58	21.87
10	80	13.87	18.95	27.41	10.20	14.05	20.65	14.20	19.30	28.08
50		9.51	15.77	24.58	6.55	11.19	18.13	9.67	16.12	25.44
**Effect of QT/RR hysteresis included**										
10	50	10.47	14.24	20.54	8.03	10.50	15.00	10.45	14.33	21.05
50		9.43	14.54	22.01	7.08	10.49	16.09	9.42	14.72	22.72
10	80	15.78	20.43	28.53	12.28	15.61	21.79	15.86	20.56	29.02
50		11.26	17.10	25.59	8.49	12.58	19.16	11.26	17.30	26.33

*Values are in milliseconds. For each formula, three columns are shown, these show the ΔQTc errors averaged over the area of initial heart rate 50 to 80 bpm and heart rate changes between -5 and +5 bpm (the left column of each formula), between −10 and −5 bpm pooled together with +5 to +10 bpm (middle column), and between −15 to −10 bpm pooled together with +10 to +15 bpm (right column). The averages are repeated for the experiments without QT/RR hysteresis influence (i.e., those shown in Figure 3–8 – top parts of the table) and for experiment with QT/RR hysteresis influence (i.e., those shown in Figure 9–12 – bottom parts of the table). The results are shown for the experiments modeling *N* = 10 and *N* = 50 study subjects and for each of these, both the averages of median (50th percentile) and of 80th percentile of the ΔQTc errors are shown. The cells with ΔQTc above 10 ms are shown in red, those with ΔQTc between 8 and 10 ms are shown in yellow.*

## Discussion

Figure 5–12 and the Table 2 show that the Fridericia and Framingham corrections lead to lesser errors compared to the other 4 correction formulae. We also found little difference between Fridericia and Framingham corrections. It appears that in larger clinical studies, such as those modeled by our experiments with *N* = 50 subjects, these formulae could be reasonably to use if the drug-induced heart rate changes do not exceed ± 10 bpm. Our results suggest that for smaller studies it might be more important to minimize the variability in QTc introduced by not accounting for QT/RR hysteresis. As already explained, the errors of QTc changes by the fixed correction depend not only on the underlying heart rate change but also on the initial heart rate.

We modeled only errors of ΔQTc data whilst in thorough QT studies, correction of the data for both baseline and placebo leads to ΔΔQTc values (Guidance to industry, 2005). This means that errors reported by our experiments have somewhat underestimated the errors that might be expected in actual studies. Hence, the greater the mean on-drug heart rate change the more caution is needed when using fixed heart rate corrections.

The method for selecting the characteristics of an individual modeling experiment needs to be discussed. Evaluation of drug-induced QTc changes is not driven by mean changes over an investigated population but by upper confidence intervals, that is, either the upper single-sided 95% confidence intervals of QTc changes in individual study time-points controlled for both baseline and placebo in the so-called intersection union test (Guidance to industry, 2005), or by the same upper confidence interval of QTc change derived from a pharmacokinetic-pharmacodynamic regression model (Garnett et al., 2008). For this reason, we used the exclusion of the low and high 10% of the errors in individual subjects of each experiment and considered the maximum absolute error of the modeled data after this exclusion. Hence, this approach approximated the extent in which the inaccurate corrections might influence the interpretation of study results. In each case (i.e., in each h and δ combination), the distribution of the errors was mostly positioned on one side of the zero line. Therefore, the exclusion of 10% of results on each end and taking the maximum absolute value after the exclusion modeled single-sided 90th percentile of the absolute errors. This reflected the practice of thorough QT study evaluations. (Note that all experiments were set-up in such a way that with accurate subject-specific corrections of heart rate and QT/RR hysteresis influence, all the characteristics would be equal to zero).

The modeling experiments investigated the differences between the fixed corrections and subject-specific QT/heart-rate curvilinear regressions. In other words, the experiments investigated the effects of fixed corrections in comparison of what can be optimally obtained from studying individual QT/heart-rate relationship and QT/RR hysteresis. Nevertheless, the QT and heart rate data that we used to derive the subject-specific regressions showed additional intra-subject QTc variability around 5 ms. This needs to be considered when interpreting the results of the experiments. The errors of QTc investigations based on fixed corrections might have been underestimated.

The differences between the medians and upper 80th percentiles of the experimental results were surprisingly small suggesting that the distribution of the experimental results was rather narrow. It thus seems reasonable to use the 80th percentile data in power calculation projections of our results. In other words, while the presentation of median results shows the errors that might be expected in “averaged” study situations, the 80th percentile shows the errors that should be considered when designing a new clinical pharmacology study (the percentile reflects the practice of 80% probability of type II error elimination).

It has been previously suggested that physiologically independent processes determine how much QT interval changes in response to the underlying heart rate, and how quickly QT interval adapts to heart rate changes (Malik et al., 2008b). This is the distinction between QT/heart-rate adaptations and QT/RR hysteresis. Surprisingly, whilst the correction of the QT interval for heart rate (i.e., for QT/heart-rate adaptation) is universally accepted, the correction for QT/RR hysteresis is frequently omitted (Malik et al., 2016). At the same time, our results show that neglecting the effects of QT/RR hysteresis decreases the accuracy of QTc assessment noticeably. It is also frequently, but falsely believed that QT/RR hysteresis needs to be considered only in cases of abrupt and substantial heart rate fluctuations, e.g., when heart rate change occurs immediately after the drug administration. Whilst in such situations, the necessity of correcting for QT/RR hysteresis is obvious (Malik et al., 2009), the need for it is not restricted to such cases. As we have previously shown, stable heart rate history of QT measurements is rare in clinical studies and thus, correction for QT/RR hysteresis should always be considered. In usual situations, there is only little difference between subject-specific corrections of QT/RR hysteresis (Malik et al., 2008b) and universal hysteresis correction (Malik et al., 2016). The universal hysteresis correction can easily be combined with fixed heart rate correction thus replicating the improvement of data accuracy demonstrated in our experiments. It should also be noted that the distribution of immediate heart rate instability (bottom panel of Figure 1) contained rather small values. Still, the modeling experiments show that even such small heart rate instabilities can have a clearly noticeable effect on the QTc data accuracy.

As already explained, absolute differences between correction formulae are potentially misleading when studying drug-induced changes. The ΔQTc values of baseline to on-treatment differences are important. The absolute differences between corrections become only of interest when relating the QTc values to an absolute threshold (e.g., 500 ms) (Fenichel et al., 2004).

Not surprisingly, our experiments with *N* = 10 subjects showed larger errors compared to those with *N* = 50. Indeed, this corresponds to the known experience that in larger studies, the increased number of QTc measurements helps reducing the confidence intervals of the mean QTc changes. Importantly, images in Figure 9, 11 show appreciable level of correction errors even in cases with heart rate change δ equal or close to zero. This is because in these cases, the spontaneous heart rate fluctuations are combined with the immediate heart rate instability. This again underlines the need for QT/RR hysteresis correction, particularly in smaller clinical studies (Malik et al., 2018).

We are not aware of other published studies with which we could compare the results of our modeling experiments. Nevertheless, consistent with other reports (Rautaharju et al., 1990), we found the Bazett formula to be the least accurate of all those tested. The experiments with the Fridericia formula confirmed the previous observations that on average, it is more accurate in males than in females (Malik et al., 2016).

Our results have main prospective value in providing an informed guidance for future investigations including the level on errors that need to be considered in power sample calculations (Zhang and Machado, 2008). Equally importantly, the modeling results identify circumstances in which the fixed corrections become potentially so problematic that it would be difficult to accept them in studies supporting regulatory submissions of new pharmaceuticals or of new treatment applications of existing drugs (Garnett et al., 2012).

### Limitations

Limitations of our statistical modeling also need to be considered. We have selected a battery of only 6 formulae while many others have also been proposed (Malik, 2002). Nevertheless, we selected these formulae to represent a wide spectrum of mathematical approaches. Also, since it has also been shown that the subject-specific slopes, curvatures, and central values of QT/heart-rate profiles are highly individual (Batchvarov et al., 2002; Malik et al., 2013), it is doubtful that any other fixed formula would clearly outperform Fridericia and Framingham corrections. The extreme settings of our modeling experiments were obviously not realistic. The broad ranges of the h and δ coefficients in our experiments were thus used for illustrative purposes. The regions of baseline heart rate and heart rate changes summarized in Table 2 are more realistic and have indeed been used when interpreting the results of the experiments. The source data we used were derived from recordings of healthy subjects. We cannot comment on the situations in patients in whom the QT/heart-rate profiles are influenced by pathological circumstances. Nevertheless, such circumstances would likely increase the individuality of the profiles and thus, if anything, further decrease the applicability of fixed correction formulae. Finally, the statistical modeling experiments were designed to correspond to cross-over (or partially cross-over) studies in which each subject is also recorded on placebo and serves as her/his own control. Parallel studies that compare different on-treatment and on-placebo populations might require different modeling approach.

### Practical Implications

Despite these limitations, we conclude that when investigating drug-induced QTc changes in the presence of drug-related heart rate changes in reasonably sized studies of healthy volunteers, the Fridericia formula might be appropriate if the heart rate change does not exceed ±10 bpm. In smaller studies, such the typical first-in-man investigations, the impact of using fixed heart rate correction as well as the impact of not accounting for QT/RR hysteresis is greater than for larger trials. The same limits of heart rate change appear applicable to Framingham formula while other fixed corrections should be avoided unless independently justified. When the drug-induced heart rate changes exceed ±10 bpm, the use of fixed heart rate QTc corrections becomes problematic and alternative methods such as subject-specific heart rate corrections might need to be considered.

## Data Availability

All datasets generated for this study are included in the manuscript.

## Author Contributions

All authors listed have made a substantial, direct and intellectual contribution to the work, and approved it for publication.

## Disclaimer

This article reflects the views of the authors and should not be construed to represent the U.S. FDA’s views or policies.

## Conflict of Interest Statement

The authors declare that the research was conducted in the absence of any commercial or financial relationships that could be construed as a potential conflict of interest.
